# Lister’s Latest Antiseptic Dressing

**Published:** 1886-08

**Authors:** 


### Medical News.

BUFFALO is the only city in this State whose vital statistics show an excess of births over deaths. Of course, there is an excess of births in all cities, but the vital statistics are so loosely kept that this is not shown. In 1884, there were 5,410 births and 3,906 deaths. The birth-rate was 27¾ per 1,000 and the death-rate 20 per 1,000. In 1885, there were 5,731 births and 3,895 deaths reported. The birth-rate was 28 per 1,000 and the death-rate 19 per 1,000. Our vital statistics show a birth-rate of over 8 per 1,000 above the death-rate. As there are some births unreported, it is probable that our birth-rate is about 10 per 1,000 in excess of the death-rate. During the last two years, there were reported 5,736 male births and 5,448 females, showing that there are 105¼ males born to every 100 females, about the usual ratio the world over. Of still-births, there are 127 males to to every 100 females.

LISTER'S LATEST ANTISEPTIC DRESSING.—Lister's latest antiseptic dressing is known as salalembroth. He uses it exclusively in his wards with fine results. It is a double mercurial salt, made by the sublimation of a mixture of perchloride of mercury and chloride of ammonium. It is very soluble, and has not been used in medicine since the time of the alchemists. All dressings—gauze, cotton-wool, bandages, lint, bedding, patients' underclothing, etc.,—are soaked in a 1 to 100 solution and dried. He colors these dressings with aniline blue, 1 to 10,000, so that when an alkaline discharge comes in contact with the dressings, the blue is removed and turns reddish, enabling him to see where the discharge has been and its quantity, however small or large, moist or dried.

